# The complete chloroplast genome of *Rhododendron shanii* W.P. Fang (Ericaceae), a endemic plant from the Southern Dabie Mountains of China

**DOI:** 10.1080/23802359.2022.2127336

**Published:** 2022-10-11

**Authors:** Huan-Xi Yu, Wang-Gu Xu, Jian-Liang Zhang, Ying-Ying Lv, Peng Chen, Bao-Kun Xu, Chang Qu, Zhi Wang

**Affiliations:** Nanjing Institute of Environmental Sciences, Ministry of Ecology and Environment of the People’s Republic of China, Nanjing, China

**Keywords:** Complete chloroplast genome, Ericaceae, phylogenetic analysis, *Rhododendron shanii*

## Abstract

*Rhododendron shanii* W.P. Fang 1983 (Ericaceae) is woody plant naturally distributed in the southwest of Anhui, China. The complete chloroplast genome sequence of *R. shanii* was generated by whole-genome next-generation sequencing data and assembled based on three *Rhododendron* species chloroplast genome. The complete chloroplast genome sequence of *R. shanii* was 204,170 bp and divided into four distinct regions: small single-copy region (2615 bp), large single-copy region (107,189 bp), and a pair of inverted repeat regions (47,183 bp). The genome annotation displayed 150 genes, including 95 protein-coding genes, 47 tRNA genes, and eight rRNA genes. Phylogenetic analysis with the Ericaceae reported chloroplast genomes revealed that *R. shanii* is sister to the clade comprising *R. delavayi*, *R. griersonianum* and *R. platypodum*.

*Rhododendron shanii* Fang (Ericaceae) is a woody plant, which is mainly distributed in the mid-subtropical hilly areas of the southwest of Anhui province in China. *R. shanii* is a narrowly distributed species in the Dabie Mountain, its distribution range is only limited to a long and narrow zone with an area of 110 km^2^ in the junction district of Yuexi County, Huoshan County in Anhui Province and Yingshan County in Hubei Province, and mainly distributes on the ridge or on the slope near the ridge at altitude over 1400 m (Zhao et al. 2012). Zhao kai believes that according to their investigation results and classification standard of IUCN endangered species red list, it is determined that the endangered level of *R. shanii* should be vulnerable grade (VU) at least (Zhao et al. 2012). The plant is used as ornamental flowers, which is endemic to the southern Dabie Mountains (Zhao et al. 2010). Here, we reported the complete chloroplast (cp) genome of *R. shanii*, deposited the annotated cp genome into GenBank with the accession numbers MW374796.

Fresh leaves of *R. shanii* were collected from Duozhijian of Yuexi County (31°3′50″E 116°10′52″E), Anhui province, China. Then these leaves were dried with the silicone. Voucher specimens were deposited at Nanjing Institute of Environmental Sciences (http://ppbc.iplant.cn/tu/9352036, Huanxi Yu and 2280328008@qq.com), and the voucher number of the specimen is DZJ202001. The genomic DNA was extracted following the modified CTAB method from the dry and healthy leaves (Doyle and Doyle 1987). The isolated genomic was manufactured to an average 400 bp paired-end (PE) library and sequenced by Illumina genome analyzer (Hiseq PE400). Sequencing service was provided by Personal Biotechnology Co., Ltd. Shanghai, China. The filtered reads were assembled using the program NOVOPlasty (Dierckxsens et al. 2017) with the complete cp genome of *Rhododendron delavayi*, *R. griersonianum* and *R. platypodum* as the reference (GenBank accession number MN413198, MT533181, MT985162 and MN711645). The assembled cp genome was annotated using Geneious 11.0.4 and corrected manually (Kearse et al., 2012). The complete cp genome of 11 species was aligned using MAFFT (Katoh et al. 2002). Total 11 species of Ericaceae were employed to build the maximum likelihood (ML) tree using RaxML (Stamatakis 2006) with 1000 bootstrap replicates. The cp genome of *R. shanii* was 204,170 bp in length (GenBank accession number MW374796), containing a large single-copy region (LSC) of 107,189 bp, a small single-copy region (SSC) of 2615 bp, and a pair of inverted repeat (IR) regions of 47,183 bp. Genome annotation predicted 150 genes, including 95 protein-coding genes, 47 tRNA genes, and 8 rRNA genes.

With released complete cp genome of Ericaceae, the phylogenetic analysis suggested that *R. shanii* is close to *Rhododendron delavayi Franch.*, *Rhododendron griersonianum Balf. f. & Forrest* and *Rhododendron platypodum Diels* (Figure 1). *Arbutus unedo* is the outgroup in the phylogenetic analysis. Our study represents the fist look into the complete cp genome of *R. shanii*, with 2001 bp longer than that of *R. delavayi* (Li et al. 2020), 2297 bp shorter than that of *R. griersonianum* (Liu et al. 2020) and 3123 bp longer than that of *R. platypodum* (Ma et al. 2021) separately. This complete cp genome can provide a genomic resource and contribute to constructing phylogenetic relationships and evolutionary studies among the subgenus *Hymenanthes* (Blume) K. Koch. and relative groups.

**Figure 1.**
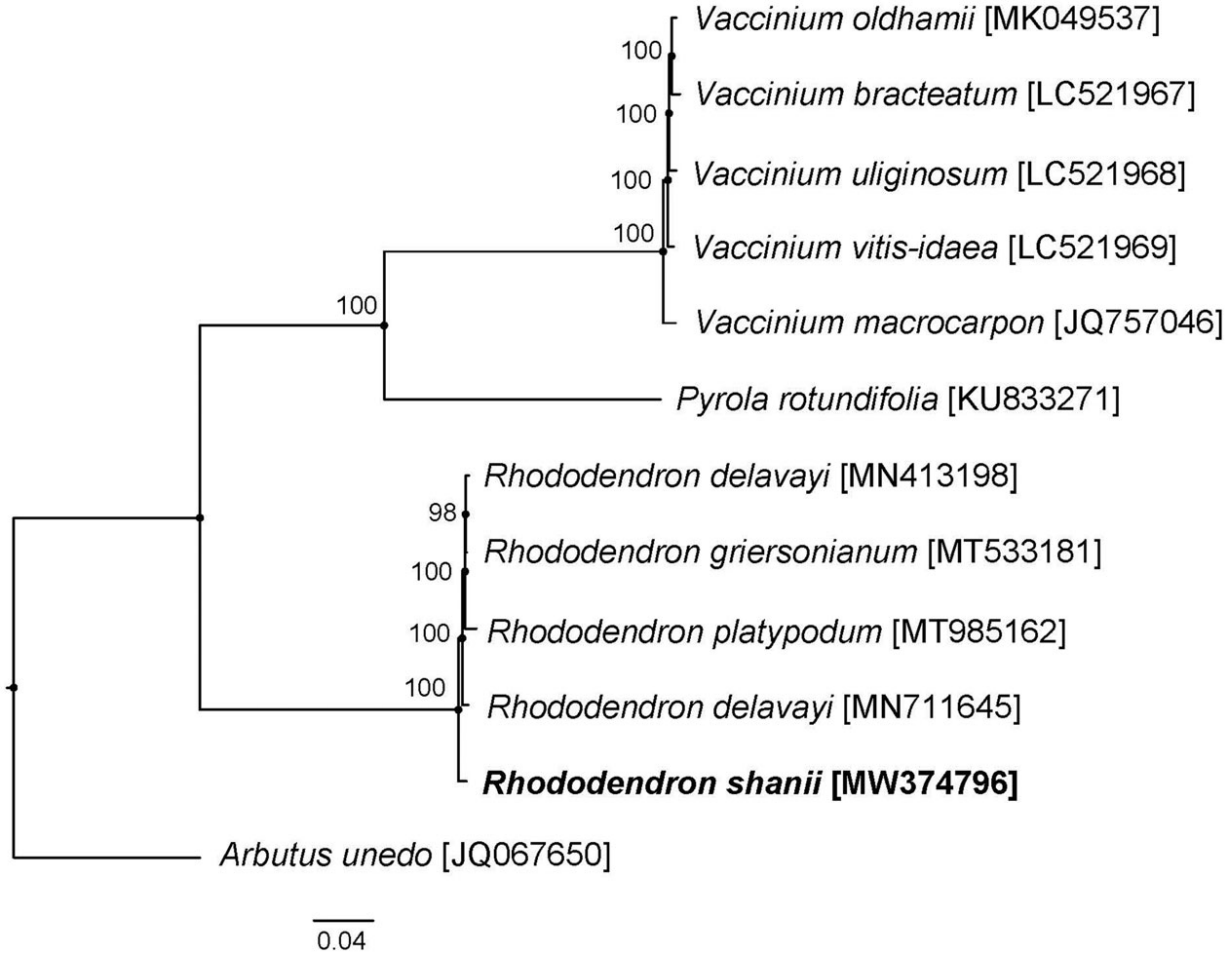
ML phylogenetic tree of *R. shanii* with 11 species of Ericaceae was constructed by chloroplast genome sequences. Numbers on the nodes are bootstrap values from 1000 replicates.

## Data Availability

The complete chloroplast genome data supporting this study have been deposited in GenBank under the accession number MW374796 and is also openly available at https://www.ncbi.nlm.nih.gov/nuccore/MW374796.1/. The associated BioProject, SRA, and Bio-Sample number are PRJNA792687, SRR17380253, and SAMN24450521, respectively.
